# Barriers to addressing mental health issues in childbearing women in Ghana

**DOI:** 10.1002/nop2.564

**Published:** 2020-08-01

**Authors:** Samuel Adjorlolo, Lydia Aziato

**Affiliations:** ^1^ Department of Mental Health School of Nursing and Midwifery College of Health Sciences University of Ghana Accra Ghana; ^2^ Research and Grant Institute of Ghana Accra Ghana; ^3^ Department of Adult Health School of Nursing and Midwifery College of Health Sciences University of Ghana Accra Ghana

**Keywords:** Africa, barriers, childbearing women, Ghana, maternal mental health

## Abstract

**Aim:**

The aim of the study is to investigate factors hampering the provision of mental health services by nurses and midwives to childbearing women to assist in the prioritization and distribution of limited mental health resources.

**Design:**

This is a cross‐sectional self‐report study.

**Methods:**

Data collected from 309 nurses and midwives were analysed using descriptive and inferential statistic, namely chi‐square and analysis of variance (ANOVA).

**Results:**

The barriers reported by the participants include unavailability of mental health services (77%), lack of knowledge of mental health in women from different tribes (75.7%), lack of a clear mental healthcare pathway (75.1%), heavy workload (74.1%) and lack of knowledge of mental health issue (74.1%). These barriers are less likely to be reported by participants who are males, old and who have practiced for a long time.

**Conclusions:**

Systematic effort to restructure the healthcare delivery system, including equipping healthcare professionals with requisite knowledge, skills and competencies in maternal mental health, is highly recommended.

## 1 INTRODUCTION

Childbearing women, defined as women in the prenatal, perinatal and postnatal periods, are at heightened risk of mental health issues such as depression and anxiety. Globally, it has been estimated that one in five childbearing women is afflicted with mental health issues (Bayrampour, Hapsari, & Pavlovic, 2018). In low‐ and middle‐income countries (LMICs), however, the prevalence rate is reportedly high, with estimates ranging from 15%–57% (Lund et al., 2014). Relating specifically to Ghana, a sub‐Saharan African (SSA) country, emerging studies have estimated the prevalence of depression in childbearing women to range from 10%–38% (Bindt et al., 2013; Weobong et al., 2014), whereas anxiety was estimated at 33% (Bindt et al., 2013). Studies have linked poor mental health issues in childbearing women to several adverse outcomes, including prolong labour and peripartum and postpartum complications (Bindt et al., 2012; Sumankuuro, Crockett, & Wang, 2017; Weobong et al., 2014). Mental health issues in childbearing women are among the factors predisposing to maternal and child morbidity and mortality rates that are reportedly high in Ghana and other SSA countries (Bindt et al., 2012; Sumankuuro et al., 2017; Weobong et al., 2014).

In recognition of the gravity of mental health issues in childbearing women, professional organizations (National Institute for Health and Care Excellence, 2014) and researchers (Siu et al., 2016) have recommended routine screening to identify women at risk of mental health problems for early intervention. However, empirical evidence suggests that most childbearing women at risk of mental health problems are undetected, undiagnosed and untreated (Anding, Röhrle, Grieshop, Schücking, & Christiansen, 2015; Bayrampour et al., 2017; Fonseca, Gorayeb, & Canavarro, 2015). This observation provides the impetus for studies to explicate factors that can be improved to promote mental and emotional well‐being in this vulnerable population. Consequently, the current study is aimed at investigating potential barriers to addressing mental health issues in childbearing women in Ghana.

## 2 BACKGROUND

A recent systematic review has identified several studies, mostly from high‐income countries, that have uncovered barriers to addressing mental health issues in childbearing women (Bayrampour et al., 2018). Notable among them are lack of knowledge in mental health, time constraint and lack of local guidelines/policies, unclear pathways of care and referral options (Bayrampour et al., 2018; Higgins et al., 2018). In SSA, however, little information exists with respect to the factors militating against the provision of mental health services to childbearing women (Bayrampour et al., 2018). Although studies conducted in high‐income countries have proffered some useful insights regarding mental health issues in childbearing women, their utilities in SSA may be limited owing largely to the sociocultural differences in healthcare systems with respect to the training of health personnel, as well as the organization and delivery of healthcare services. For instance, relative to high‐income countries, SSA countries have few qualified (mental) health professionals (Dussault & Franceschini, 2006; Labonté, Packer, & Klassen, 2006) and insufficient budgetary allocation to meet the health needs of the populace (Raja, Wood, de Menil, & Mannarath, 2010). Additionally, because most of existing studies from high‐income countries are qualitative in nature (Bayrampour et al., 2018), little is known about the importance practitioners attach to the various potential barriers (Higgins et al., 2018). Quantifying potential barriers has the propensity to unearth the enormity of the problem, expand our understanding of the problem and more importantly help in the design of context‐specific interventions (McEvoy et al., 2014). Indeed, although previous studies have advocated for continuous professional development programmes for health professionals to acquire the requisite mental health competencies (Bayrampour et al., 2018; Higgins et al., 2018), such programmes can be tailored appropriately by understanding health professionals' perceptions of potential barriers in relation to their local context (Hauck et al., 2015). Consequently, the overarching goal of the present study is to investigate the barriers influencing the delivery of mental health services to childbearing women in Ghana. The study addressed the following research question: What are the barriers and factors influencing the delivery of mental health services to childbearing women in Ghana?

### 2.1 Objectives of the study

1. Quantify the factors that midwives and primary care nurses (General Nurses and Public Health Nurses) perceived as affecting their efforts to promote mental health issues in childbearing women.
2. Investigate the extent to which demographic variables, namely age, gender, duration of practice and professional category (midwives and primary care nurses) influence barriers to addressing maternal mental health concerns.

## 3 THE STUDY

### 3.1 Design and data collection procedure

The study design and data collection procedure has been described elsewhere (Adjorlolo, Aziato, & Akorli, 2019). Briefly, a cross‐sectional self‐report survey was used to gather data from the participants across the various facilities after institutional permission was obtained from management. The participants were recruited from antenatal, delivery and postnatal units of their respective health institutions using both convenience sampling approach. The research team approached nurses and midwives on the various duty shifts (i.e. morning, afternoon and night) for their consent to participate in the study. They were informed that their participations in the study will help in understanding strategies to promote mental health issues in childbearing women. Because heavy workload at the facilities could interfere with the data collection process, effort was made to administer the questionnaires when the facilities were less busy, in this case mostly in the afternoon. They were given time to ask questions or raise issues they have with the research. Those who expressed willingness and consented to be part of the study were handed a pack of questionnaires containing several measures, including those measuring barrier and demographic variables. The questionnaires were handed over to the research team on completion. Of the 320 questionnaires administered, 309 were returned, representing a response rate of 97%.

### 3.2 Setting and participants

Data for this study form part of a large‐scale study investigating strategies to promote mental health in childbearing women in Ghana (Adjorlolo et al., 2019). Data were collected from 309 primary care nurses and midwives recruited from randomly selected health facilities that are highly patronized by childbearing women in Greater Accra, the capital of Ghana. These include Pentecost Hospital, Abokobi Health Center and University of Ghana hospital. To be included in the study, the participants must be fully registered with the appropriate licensing authority (i.e. Nursing and Midwifery Council) and should have worked with childbearing women for at least 12 months, a period within which the participants might have encountered childbearing women with or in need of mental health services. A large proportion of the participants were females (*N = *296, 96%). The participants designated as young (i.e. 21–30 years) were 140 (45.3%) whereas those designated as old (i.e. 31 years and above) were 169 (54.7%). A large proportion were females (*N = *296, 96%), public health nurses (*N = *138, 44.7%), general nurses (88, 28.5%) and midwives (83, 26.9%). The percentage of participants who reportedly practiced for 1–5 years and 6 years and above were 174 (56.3%) and 135 (43.7%), respectively. In terms of qualification, most participants had diploma (i.e. 3 years of nursing education; *N = *171, 55.3%), followed by certificate (i.e. <3 years of nursing education; *N = *116, 37.5%) and degree or advanced qualification (i.e. more 3 years of nursing education; *N = *22, 7.1%).

### 3.3 Measures and instrument

The participants' responses to measures of demographic variables namely gender, age, years of practice and professional category and barrier items were used in the current study. The barrier items, culled predominantly from the existing literature (Bayrampour et al., 2018; Higgins et al., 2018), were included in the study on the recommendation from health practitioners (e.g. midwives) and scholars in Ghana. A total of 25 barrier items (shown in Table 1) were used in this study. In keeping with the response format of existing studies (Higgins et al., 2018), the items were scored on a 4‐point Likert‐type scale, ranging from 1 (i.e. strongly disagree)–4 (strongly agree). The total score on the “barrier scale” ranges from 28–100, with high score indicating high perceived barriers. The internal consistency of the “barrier scale” was 0.88.

**TABLE 1:** Barriers to discussing mental health in childbearing women

	All Midwives/Nurses		Yes			Yes		
	No	Yes	Midwives *N = *83	Practice Nurses *N = *88	Public Health Nurses *N* = 138	Certificate *N = *116	Diploma *N = *171	Degree *N = *22
	*n* (%)	*n* (%)	*n* (%)	*n* (%)	*n* (%)	*n* (%)	*n* (%)	*n* (%)
Heavy workload	80 (25.9)	229 (74.1)	59 (71.1)	64 (72.7)	106 (76.8)	87 (75)	125 (73.1)	17 (77.3)
Time allocated too short	110 (35.6)	199 (64.4)	51 (61.4)	56 (63.6)	92 (66.7)	75 (64.7)	107 (62.6)	17 (77.3)
No clear mental healthcare pathway	77 (24.9)	232 (75.1)	59 (71.1)	69 (78.4)	104 (75.4)	90 (77.6)	125 (73.1)	17 (77.3)
Lack of knowledge of perinatal mental health issue	80 (25.9)	229 (74.1)	54 (65.1)	70 (79.5)	105 (76.1)	91 (78.4)	122 (71.3)	16 (72.7)
Lack of knowledge on perinatal mental health and women from different tribes	75 (24.3)	234 (75.7)	56 (67.5)	73 (83)	105 (76.1)	89 (76.7)	126 (73.7)	19 (86.4)
Not seeing women regularly to build relationship	95 (30.7)	214 (69.3)	55 (66.3)	67 (76.1)	92 (66.7)	84 (72.4)	114 (66.7)	16 (72.7)
Perinatal mental health services not available	71 (23.0)	238 (77.0)	65 (78.3)	69 (78.4)	104 (75.4)	93 (80.2)	130 (76)	15 (68.2)
No organizational structure/process to see women alone	106 (34.3)	203 (65.7)	52 (62.7)	58 (65.9)	93 (67.4)	72 (62.1)	114 (66.7)	17 (77.3)
Lack skill to respond to disclosure of issue	156 (50.5	153 (49.5)	45 (54.2)	36 (40.9)	72 (52.2)	54 (46.6)	89 (52)	10 (45.5)
Isolated from knowledgeable colleagues	193 (62.5)	116 (37.5)	32 (38.6)	38 (43.2)	46 (33.3)	56 (48.3)	53 (31)	7 (31.8)
Fears that women could misinterpret questions as a judgement	139 (45.0)	170 (55.0)	44 (53)	50 (56.8)	76 (55.1)	68 (58.6)	93 (54.4)	9 (40.9)
Uncertain of whether women want to be asked about MH issues	119 (38.5)	190 (61.5)	52 (62.7)	52 (59.1)	86 (62.3)	69 (40.5)	111 (64.9)	10 (45.5)
Fears that women could get offended	121 (39.2)	188 (60.8)	55 (66.3)	47 (53.4)	86 (62.3)	71 (61.2)	108 (63.2)	9 (40.9)
Fears that women could get emotionally distressed when discussing their MH	107 (34.6)	202 (65.4)	57 (68.7)	58 (65.9)	87 (63)	73 (62.9)	118 (69)	11 (50)
Discomfort discussing MH issues with woman if partner/family/support person present	136 (44.0)	173 (56.0)	53 (63.9)	42 (47.7)	78 (56.5)	60 (51.7)	100 (58.5)	13 (59.1)
Lacks knowledge of how to access MH services/supports for women	125 (40.5)	184 (59.5)	48 (57.8)	50 (56.8)	86 (62.3)	74 (63.8)	98 (57.3)	12 (54.5)
Lacks authority to discuss MH issues with women	148 (47.9)	161 (52.1)	40 (48.2)	44 (50)	77 (55.8)	66 (56.9)	87 (50.9)	8 (36.4)
Lacks support from colleagues or managers if a mental health issues emerges	140 (45.3)	169 (54.7)	43 (51.8)	48 (54.5)	78 (56.5)	75 (64.7)	82 (48)	12 (54.5)
Fear that if a woman referred the to the psychiatrist she will only receive medication	199 (64.4)	110 (35.6)	29 (34.9)	31 (35.2)	50 (36.2)	48 (41.4)	56 (32.7)	6 (27.3)
Concern that their relationship with women would be negatively affected	205 (66.3)	104 (33.7)	31 (37.3)	24 (27.3)	49 (35.5)	41 (35.3)	54 (31.6)	9 (40.9)
Fears that women think that discussing MH issues is not the role of the nurse/midwife	207 (67.0)	102 (33.0)	27 (32.5)	25 (28.4)	50 (36.2)	43 (37.1)	54 (31.6)	5 (22.7)
Documenting mental health issues would stigmatize the woman	206 (66.7)	103 (33.3)	29 (34.9)	32 (36.4)	42 (30.4)	45 (38.8)	53 (31)	5 (22.7)
Discussing MH is a taboo subject	242 (78.3)	67 (21.7)	22 (26.5)	19 (21.6)	26 (18.8)	25 (21.6)	37 (21.6)	5 (22.7)
Talking about MH could increase the risk of self‐harm/suicide	239 (77.3)	70 (22.7)	22 (26.5)	19 (21.6)	29 (21)	28 (24.1)	37 (21.6)	5 (22.7)
Talking about MH could increase the risk of harm to the baby	237 (76.7)	72 (23.3)	21 (25.3)	20 (22.7)	31 (22.5)	26 (22.4)	41 (24)	5 (22.7)

Note

*N = *309; No = Strongly disagree + Disagree; Yes = Strongly agree + Agree.

### 3.4 Data analytic strategy

All data were analysed with SPSS Version 23 (IBM.corp). Cases with missing data on the barrier items represent less than 1% of the total cases, and because data were missing completely at random (Little's MCAR chi‐square > 0.05), the expectation maximization algorithm was used to impute the missing data points. A two‐tailed statistical significance was set at 0.05. To ascertain the usefulness of each of the 25 barrier items, the corrected item‐total correlations, squared multiple correlations (SMCs) and Cronbach's alpha when items were deleted were examined. The results indicate that the items are measuring barrier to the provision of mental health services to childbearing women. Responses on the barrier items were subsequently dichotomized to reflect No (i.e. Strongly Disagree and Disagree) and Yes (i.e. Strongly Agree and Agree). This was subjected to descriptive statistics, namely frequency and percentages, to determine the proportion of “No” and “Yes” responses. Chi‐square was used to investigate the bivariate relationship between the barrier items and demographic variables (i.e. age, gender, years of practice and education), with effect size estimated with Phi and Cramer's *V* coefficients. Additionally, the 25 barrier items, rated on the four‐point Likert response format, were subjected to principal component analysis (PCA) to determine their underlying latent component(s). Analysis of variance (ANOVA) was subsequently conducted to determine the effects of demographic variables, using the extracted component(s) as the dependent variable.

## 4 RESULTS

### 4.1 Barriers to addressing mental health issues in childbearing women

The results of the analyses regarding the barriers to addressing mental health issues in childbearing women are summarized in Table 1. As can be seen, the participants attached different weights to the various barrier items. The top most rated barriers are unavailability of mental health services (*N = *238, 77%), lack of knowledge on mental health and women from different tribes (*N = *232, 75.7%), lack of a clear mental healthcare pathway (*N = *232, 75.1%) and heavy workload (*N = *229, 74.1%) and lack of knowledge of mental health issue (*N = *229, 74.1%). Other barriers endorsed by more than half of the participants include not seeing women regularly to build relationship (*N = *214, 69.3%), no organizational structure or process to see women alone (*N = *203, 65.7%), fears that childbearing women could get emotionally distressed when discussing mental health issues (*N = *202, 65.4%), the short time allocated to seeing childbearing women (*N = *199, 64.4%), uncertainty about whether women want to be asked about mental health (*N = *190, 61.5%) and fears that women could get offended (*N = *188, 60.8%). Table 1 further shows that the rating of the barrier items appeared not to differ in any systematic way based on the category of profession (e.g. midwives versus PHNs) and qualification (e.g. diploma versus degree).

### 4.2 Demographic correlates of barriers

For simplicity, the demographic variables that correlate significantly with the barriers to addressing mental health issues in childbearing women are presented here (the full result is available on request). First, increasing age was associated with a decrease in perceiving the following as barrier items: talking about mental health could increase the risk of self‐harm/suicide, [*χ* ^2^ = 3.96, *p *= .047, Phi & Cramer's *V* = 0.11] and talking about mental health could increase the risk of harm to the baby, [*χ* ^2^ = 3.98, *p *= .046, Phi & Cramer's *V* = 0.11]. Relatedly, being a female increases the likelihood of rating the following as barriers: uncertainty about whether women want to be asked about mental health issues [χ^2^ = 5.41, *p *= .020, Phi & Cramer's *V* = 0.13] and lack of authority to discuss mental health issues with women [*χ* ^2^ = 4.58, *p *= .032, Phi & Cramer's *V* = 0.12]. Lastly, a significant relationship was found between years of practice and the following barriers: not seeing women regularly to build relationship, [*χ* ^2^ = 4.5, *p *= .035, Phi & Cramer's *V* = 0.12] and lack skill to respond to disclosure of issue [*χ* ^2^ = 5.10, *p *= .024, Phi & Cramer's *V* = 0.13]. The result suggests that participants who have practiced for 6 years and above are less likely to rate the above as barriers.

### 4.3 Principal component analysis and effects of demographic variables on barriers

As noted previously, PCA was conducted to investigate possible clustering of the barrier items. The results of the initial PCA showed that the clustering of the barrier items did not yield any interpretable component. Consequently, based on the presumption that all the items reflect a single underlying construct (i.e. barrier to delivering mental health services to childbearing women) and in the interest of scientific parsimony, one component was extracted. The results of the PCA suggested no problem with multicollinearity, given that the determinant of matrix of 8.75 was greater than necessary value of 0.00001 (Field, 2013). The Kaiser–Meyer–Olkin measure of 0.85 and Bartlett's test of sphericity, *χ* ^2^ (300) = 2,792.13, *p *< .001 showed sampling adequacy and sufficient inter‐item correlations for PCA, respectively. The eigenvalue for the extracted component was 6.57 (56.30%). This was saved for further analysis using the Bartlett method that produces unbiased scores (Field 2013). The items exhibited satisfactory factor loadings, ranging from 0.31 (heavy workload)–0.62 (fears that women could get emotionally distressed when discussing their mental health). ANOVA results revealed no statistically significant mean‐level effect of the demographic factors on barrier to delivering mental health services to childbearing women (*p *> .05; the results are available on request from first author).

## 5 DISCUSSION

In Ghana and other low‐ and middle‐income countries, there are notable challenges besetting the provision of mental health services to different populations, including childbearing women. However, studies quantifying the barriers to addressing mental health issues in childbearing women from the perspective of health professionals are notably scarce. This is the first study in Ghana and perhaps in SSA to have quantified these barriers as well as investigated the impact of demographic variables on the self‐reported barriers. It emerged that the participants endorsed several barriers to the provision of mental health services, including unavailability of mental health services, lack of a clear mental healthcare pathway and heavy workload. The finding largely supports existing studies from high‐income countries (Bayrampour et al., 2018; Higgins et al., 2018) although the challenges are more pervasive in Ghana owing to funding and logistics constraints, as discussed below.

Relating to the unavailability of mental health services, it is interesting to note that the provision of mental health services in Ghana is largely concentrated at the three public psychiatric hospitals in the country. Unfortunately, recent efforts to decentralize mental health service delivery in the country have been limited largely to regional and teaching hospitals. Importantly, because several health facilities located in urban and rural areas of the country are yet to boast of psychiatric units, it follows that mental health services are not readily available to childbearing women visiting these facilities. Similarly, the limited coordination among healthcare institutions and units or departments in Ghana (Adjorlolo, 2015) suggests that even in health facilities with psychiatric units, there is the possibility that midwives and primary care nurses may be unaware of their existence. In addition is the lack of a structured protocol detailing the pathway of addressing mental health issues in childbearing women. This development could put health professionals in a helpless situation whereby they are not able to and adequately use any existing mental health services effectively, including referral to psychiatric hospitals (Jomeen, Glover, Jones, Garg, & Marshall, 2013).

Additionally, heavy workload has been rated among the major barriers to the delivery of mental health services to childbearing women. In this regard, previous studies have shown that heavy workload limits health professionals ability to form relationships with women to provide individualized care and meet their psychological and emotional needs (Elliott, Ross‐Davie, Sarkar, & Green, 2007; Jones, Creedy, & Gamble, 2012). Heavy workload in Ghanaian setting appears to result primarily from the inadequate number of healthcare providers in general and midwives and primary care nurses in particular. For example, according to the statistics from the Ghana Health Service (GHS), the average nurse–population ratio in Greater Accra from 2008–2016 was 1:764 (GHS, 2017). The disproportionate number of patients to nurses and midwives could create a situation whereby these professionals may have to shorten their encounters with patients and clients in the quest to attend and possibly address the health needs of the large number of patients at the facilities. This would ultimately limit the opportunity to engage childbearing women in discourses pertaining to their mental and emotional well‐being, especially when the women do not show overt and debilitating symptoms of mental illness, which in the Ghanaian context are granted significant attention as signalling seeking help from professionals (Adjorlolo, 2016). Consistent with previous studies (Borglin, Hentzel, & Bohman, 2015), heavy workload could be invoked to explain the initial observation of an ongoing study in Ghana that several information on antenatal records such as mental health is either not solicited for, or entered into the records, resulting in substantial loss of salient information that can enhance individualized care.

Again, lack of knowledge on mental health in women from different tribes in particular and lack of knowledge of mental health in childbearing women in general have been rated highly by the participants. This is largely consistent with Bayrampour et al. (2018) who, in their systematic review, found that health professionals' lack of knowledge in mental health issues in childbearing women, including symptoms and risk factors, is among the common barriers to promoting mental well‐being in the women. The participants' endorsement of lack of knowledge in this study could stem predominantly from the nature of training received by the midwives and primary care nurses. In particular, a critical examination of the curriculum for training primary care nurses and midwives designed by the Nurses and Midwives' Council of Ghana revealed that little attention has been granted to mental illness as an essential subject or topic. Thus, nurses and midwives complete their respective training with no or limited knowledge in mental health in general and mental health particularly in childbearing women. Additionally, the numerous continuous professional programmes organized by the various professional bodies for nurses and midwives rarely centre on the knowledge acquisition of competencies relating to, for instance, screening and identification of mental health issues in patients and clients attending non‐psychiatric hospitals, as well as the provision of low‐cost mental health interventions such as psychoeducation. Granted the above, it is plausible to state that primary care nurses and midwives may be similar with respect to their knowledge‐based and competencies in mental health in childbearing women. This line of reasoning appeared to be corroborated by the finding of statistically insignificant mean‐level effects of demographic variables on barriers to the provision of mental health services to childbearing women. These observations largely attest to the attitude towards the organization and delivery of mental health services in Ghana. In particular, stakeholders and policymakers have and continue to pay insignificant attention to mental health services (Ofori‐Atta, Read, & Lund, 2010).

Closely related to the above is the endorsement of lack of knowledge of mental illness in women from different tribes. A similar concern, referred to as cultural competency issue, has been noted by previous studies conducted in high‐income countries (Almond & Lathlean, 2011; Borglin et al., 2015; Nithianandan et al., 2016). Relating specifically to Ghana, there are over 46 different tribes and cultures in Ghana, suggesting that the country is not necessarily culturally homogenous (Adjorlolo, Abdul‐Nasiru, Chan, & Bambi, 2018). Because cultures tend to differ with respect to socialization, worldviews and beliefs, there is the possibility that the acquisition and expression of mental health issues may vary as a function of different cultures (Adjorlolo, Abdul‐Nasiru, Chan, & Bentum, 2017; Yamagishi, Hashimoto, & Schug, 2008). More importantly, because the conceptualization of mental illness may differ from culture to culture, health professionals with dissimilar cultural backgrounds with their clients or patients may have difficulty for instance, initiating a conversation around mental and emotional problems (Almond & Lathlean, 2011; Nithianandan et al., 2016). They may also have doubt about whether a particular behaviour pattern should be granted attention as a potential mental health issue. Unfortunately, empirical studies are yet to explicate cultural influences on mental health issues in Ghana, including cultural differences in the prevalence and incidence rates of mental illness, disclosure and conceptualization of mental illness.

### 5.1 Implications for practice and research

The findings reported here have enormous implications for clinical practice and research. Relating to the former, the study calls for several initiatives, including systematic efforts to equip midwives and primary care nurses to deliver mental health services to childbearing women. The ongoing decentralization of mental health services efforts should be intensified to ensure their availability at the various health facilities. Where necessary, awareness of the existence of such services should be created among midwives and primary care nurses. Additionally, there is the pressing need to develop a protocol detailing the pathway for assessment, the provision of low‐cost mental health intervention and referral for “advanced” treatment from the psychiatric hospitals. The study further calls for resourcing and empowering midwives and primary care nurses to acquire the skills and competencies in mental health in general and mental health in childbearing women in particular. This can be achieved, for instance, via the inclusion of mental health courses as an integral component of the training manual for midwives and primary care nurses. The training programmes, including continuous professional development programmes, should also take into consideration the demographic backgrounds of the professionals, as well as cultural differences in discourses pertaining to the assessment, diagnosis and treatment of mental health issues in childbearing women. In particular, the study has shown that young nurses and midwives may not initiate conversation centring on mental health because of fear that doing so may harm the baby or predispose the women to suicide/harm. Relatedly, females are less likely to exude confidence and authority in relation to mental health discourses in childbearing women. These preliminary observations necessitate a needs‐based assessment to ensure that continuous professional developments are tailored appropriately to their mental health learning needs.

It is also recommended that the government of Ghana and other stakeholders in the provision of healthcare in Ghana take the necessary steps to reduce the nurse–population ratio by recruiting more health professionals to reduce the workload of the few nurses and midwives. This would make it relatively easier for midwives and primary care nurses to devote some amount of their time and attention to addressing mental and emotional well‐being of childbearing women.

The study has suggested several areas of empirical research in the quest to promote the provision of mental health services to childbearing women. This includes investigations of the possible impacts of cultural tendencies on the distribution, manifestation, assessment, diagnosis and treatment of mental health issues in childbearing women from different cultural background. Given the indication of negative attitudes towards mental illness among mental health professionals in Ghana (Adjorlolo et al., 2018), it is also recommended that future studies investigate the attitudes of midwives and primary care nurses towards mental health in childbearing women. Likewise, studies should investigate culturally appropriate strategies to promote positive attitudes towards mental health in child bearing. Lastly, it would be informative to determine the extent to which other factors not investigated in this study, such as geographic location and type of health facility (i.e. government versus mission), could potentially hinder the provision of mental health services to childbearing women.

### 5.2 Limitations of the study

The relatively small sample size and the use of non‐probability sampling limit the applications of the findings beyond the participants of the study. The use of only questionnaire does not provide mechanism for checking the accuracy or biasness of the responses gathered from the participants. The above notwithstanding, the findings reported here do not differ systematically from those reported by previous studies, as noted previously.

## 6 CONCLUSION

This study has found that there are barriers to midwives and primary care nurses addressing mental health concerns in childbearing women, including heavy workload, lack of clear pathways and lack of knowledge about mental health in childbearing women. Prioritizing limited resources to mitigating the impacts of the barriers reported in this study could help equip midwives and primary care nurses to render basic and low‐cost mental health services to childbearing in need of these services.

## CONFLICT OF INTEREST

The authors declare that they have no competing interests.

## AUTHOR CONTRIBUTIONS

SA collected and analysed data. LA wrote the manuscript.

## ETHICAL APPROVAL

The study received ethical clearance from the Institutional Review Board of Noguchi Memorial Institute for Medical Research, University of Ghana (NMINR‐IRB CPN 088/17‐18). Written consent was obtained in accordance with the prescriptions of the ethical clearance.
